# THE GLOBAL PRIDE STUDY: TRANSFORMATIONS IN SEXUALITY, GENDER, AND AGE IN LATER LIFE

**DOI:** 10.1093/geroni/igae098.1087

**Published:** 2024-12-31

**Authors:** Karen Fredriksen-Goldsen

**Affiliations:** Chair

## Abstract

Transformations in sexuality and gender are changing the context of global aging. The Global Pride Network and Study, involving over 50 scholars worldwide, aims to comprehensively address health and well-being in sexual and gender diverse populations, covering quality of life, physical and mental health, and economic and social well-being. This symposium will highlight the international development and implementation of the Global Pride Study across six world regions, sharing knowledge, experiences, and collaborations in studying sexuality, gender and age as they relate to well-being and health, as well as the impact of COVID-19 across diverse global communities. Fredriksen-Goldsen and colleagues will begin by presenting the project goals, international research collaborations to develop and distribute a pilot survey across global regions, and cross-cultural adaptations of research methods necessary for the implementation of multi-faceted and multi-level research strategies. Next, Oswald and colleagues will present a video of international collaborators as they evaluate the state of sexuality and gender research and policy across the globe. The gaps in the existing knowledge base and future research directions will be discussed. The final presentation, delivered by Hughes and colleagues, will present quantitative findings from the global survey of one country, Australia, investigating loneliness across the lifespan and its interaction with other life experiences. This groundbreaking symposium will contribute to advancing research addressing sexuality, gender, aging, longevity, and well-being globally, contributing to international dialogue on reframing these constructs and presenting next steps and best practices learned through this highly innovative and collaborative research process. Rainbow Research Group Interest Group Sponsored Symposium

